# Serologic testing of randomly selected children after hepatitis B vaccination: a cross-sectional population-based study in Lao People’s Democratic Republic

**DOI:** 10.1186/s12879-019-4086-0

**Published:** 2019-06-10

**Authors:** Masataro Norizuki, Tomomi Kitamura, Kenichi Komada, Masaya Sugiyama, Masashi Mizokami, Anonh Xeuatvongsa, Vilasak Som-Oulay, Phengta Vongphrachanh, Munehito Machida, Koji Wada, Koji Ishii, Tomoko Kiyohara, Takaji Wakita, Masahiko Hachiya

**Affiliations:** 1 Bureau of International Health Cooperation, National Center for Global Health and Medicine, Shinjuku, Tokyo, Japan; 2 Graduate School of Medicine, International University of Health and Welfare Graduate School, Minato, Tokyo, Japan; 3 The Research Center for Hepatitis and Immunology, National Center for Global Health and Medicine, Ichikawa, Chiba, Japan; 4 National Immunization Program, Ministry of Health, Lao PDR, Simeuang Road, Vientiane, Lao PDR; 5 National Center for Laboratory and Epidemiology, Ministry of Health, Lao PDR, Simeuang Road, Vientiane, Lao PDR; 6 Department of Global Health, Faculty of Medicine, Institute of Medical, Pharmaceutical and health Sciences, Kanazawa University, Kanazawa, Ishikawa Japan; 7 Department of Virology II, National Institute of Infectious Diseases, Musashi-murayama, Tokyo, Japan

**Keywords:** Hepatitis B, Vaccine, Post-vaccination serologic testing, Cold chain, Dried blood spot, Children

## Abstract

**Background:**

Population immunity against hepatitis B virus (HBV) in Lao People’s Demographic Republic (PDR) has not been examined since the national HBV vaccination program was started in 2002. Vaccine has been observed to be frozen at times during cold-chain transport in vaccination programs in Lao PDR and other developing countries, which will inactivate the vaccine. Therefore, this study used post-vaccination serologic testing to evaluate the effects of HBV immunization in Lao PDR.

**Methods:**

A cross-sectional serologic study was conducted among children (age range, 5–9 years) and mothers (15–45 years) who were randomly selected using probability-proportional-to-size sampling from central Lao PDR. Blood samples were collected as dried blood spots (DBS) and analyzed using chemiluminescent microparticle immunoassay to detect anti-hepatitis B surface (HBs) titers. We also evaluated the correlation between anti-HBs levels measured in DBS and serum among healthy healthcare workers in Vientiane.

**Results:**

Anti-HBs titers from DBS were strongly correlated with serum levels (correlation coefficient = 0.999) in all 12 healthcare workers evaluated. A linear regression model showed that 10 mIU/mL of serum anti-HBs was equivalent to 3.45 mIU/mL (95% CI: 3.06–3.85) of DBS. Among 911 mother-child pairs tested, 171 children had documentation of vaccination. Of the 147 children who had received ≥3 doses of the hepatitis B vaccine, 1 (0.7%) was positive for anti-HBs. The remaining 24 children received the hepatitis B vaccine only twice, once or no dose.

**Conclusions:**

The results showed extremely low positivity for anti-HBs among vaccinated children in central Lao PDR. Therefore, post-vaccination serologic testing is important to evaluate population immunity against HBV infection. DBS testing is a potential low-cost tool to evaluating the effectiveness of HBV vaccination programs.

## Background

Among the more than two billion people infected with hepatitis B virus (HBV) worldwide, in 2015, WHO estimates 257 million have chronic HBV infection [1]. The probability of developing chronic HBV infection depends on age at infection: about 90% of infants will develop chronic infection when infected within the first year of life, compared with 30% of children aged 1–4 years and less than 5% of adults [2]. Therefore, preventing HBV infection with vaccination during infancy is of paramount importance. The World Health Organization (WHO) aims to achieve more than 90% third-dose coverage for HBV vaccination by 2020 [1].

In Lao People’s Democratic Republic (PDR), the national immunization program for HBV vaccine has been underway since 2002. Three doses of recombinant vaccine were administered at age 6, 10, and 14 weeks until 2004, when birth doses were first started in the capital, Vientiane. The third-dose coverage rate of HBV among infants was around 50% during 2001 and 2007, then gradually increased and reached 78.3% by 2011 [3]. The prevalence of chronic HBV infection (i.e., the presence of HBV surface antigen (HBsAg) in the serum) before starting the HBV vaccination program was found to be 8.7% among male and female adult blood donor subjects [4], and 2.9% [5] and 4.1% [6] among women of child-bearing age. The differences in prevalence was considered to be due to sampling design and population studied [5, 6]. After the HBV vaccination program started, the prevalence of HBV infection, determined using a rapid test kit, in a nationwide cross-sectional study carried out in 2012 involving randomly sampled children aged 5–9 years was 1.7% (95% CI: 0.8–2.6%) [5]. Using dried blood spot (DBS) analysis in 2011, we found a similar prevalence of 2.1% (95% CI: 0.8–3.4%) among randomly selected children of the same age in central Lao PDR, an area which includes Vientiane and remote rural areas [6]. Mathematical modeling using data on immunization coverage and hepatitis B surface antigen (HBsAg) prevalence revealed that HBV vaccinations may have saved 22,269 children born between 1990 and 2014 from chronic HBV infection [7].

However, during our involvement in vaccination programs in Lao PDR and other developing countries, we and other teams have observed cases in easy- and hard-to-reach areas where vaccine was inadvertently frozen during cold-chain transport, which will inactivate the vaccine. In Lao PDR, these observations have been confirmed by temperature logger data [8]. This has led us to question the effectiveness of the HBV vaccination program there, and we sought to evaluate this with the present study.

Vaccination coverage is a conventional indicator of the effectiveness of vaccination programs, but when the vaccine is not active and thus cannot protect against HBV infection, another indicator is needed to measure the quality of the program. Post-vaccination serologic testing is one solution to this. However, this requires centrifugation and adequate maintenance of reverse cold chain, which results in costly investigations, and any problems of temperature control in the reverse cold chain process will compromise the testing method for positivity. To date, studies in Thailand [9] and China [10] have evaluated HBV vaccination programs using post-vaccination serologic testing, but no population-based serologic evaluations have been conducted among randomly selected children living in Lao PDR, including those in remote rural areas.

To evaluate the effectiveness of the HBV vaccination program in Lao PDR, in this cross-sectional study, we measured anti-hepatitis B surface (HBs) among randomly selected children in easy- and hard-to-reach areas in central Lao PDR, using DBS as a cost-effective method that allows for easy transportation of blood samples, especially from hard-to-reach areas. We also investigated the correlation and evaluated the cutoff values of anti-HBs titers between paired DBS and serum samples collected from healthy healthcare workers who had received ≥3 doses of HBV vaccine.

## Methods

### Participants

The participants were children aged 5–9 years and their mothers (age, 15–45 years) from central Lao PDR who were randomly selected in January 2011 using probability proportional to size (PPS) sampling. Adoption is common in rural Lao PDR, so the children were required to be biologically related to their mothers. We decided the target age of children for this study in accordance with a previous study on the seroprevalence of chronic HBV infection [6].

### Sampling

We used multistage cluster sampling to identify participants. We enrolled participants from Vientiane Municipality and four rural provinces in central Lao PDR, which accounts for 40% of the national population. The population of Vientiane Municipality was 691,721, while that of the provinces of Borikhamxay, Khammuane, Vientiane, and Savannakhet was 225,301, 337,390, 388,895, and 825,902, respectively. These five locations were considered a stratum. In the first stage of sampling, based on population data, we randomly selected four districts each from Vientiane Municipality and the four provinces using PPS sampling. In the second stage, again using PPS, we randomly selected two villages from each selected district. In the third stage, we obtained a list of households from each selected village that included children aged 5–9 years. From that list, we randomly selected 24 households using a paper-based lottery system. If a household had multiple children aged 5–9 years, we selected the youngest child [6].

### Data collection

We administered a brief face-to-face questionnaire with the mothers that gathered data on the children’s sociodemographic characteristics. We also collected documented HBV vaccination histories from immunization cards.

For blood collection, we used capillary whole blood as DBS (Whatman 903 protein saver cards; GE Healthcare, Westborough, MA) to guarantee a good quality of blood samples taken from children in hard-to-reach areas especially. After sample collection, DBS were air-dried for at least 60 min and then stored in plastic bags at ambient temperature in Lao PDR for 7–10 days before transportation to Japan, where they were then stored at 4 °C for 3 months before being tested at the Research Center for Hepatitis and Immunology, National Center for Global Health and Medicine, Ichikawa, Japan. Blood samples were extracted from the DBS by punching two bloodstained circles (diameter, 3 mm) and eluting overnight in 500 μL of phosphate-buffered saline (pH 7.2). Eluates for anti-HBs and HBsAg were tested using a chemiluminescent microparticle immunoassay (Architect i2000SR; Abbott Diagnostics, IL), and an automated system was used to detect the relative light unit (RLU) value of each sample. The sample was considered positive for HBsAg based on comparisons with the RLU value of a calibration sample [6].

### Evaluation of DBS analysis

In an additional study, we investigated the correlation and cutoff values of anti-HBs titers between serum and DBS. We performed serologic assays of anti-HBs by DBS compared with serum as the gold standard. We recruited participants from healthy volunteer healthcare workers in Vientiane Municipality who had received ≥3 doses of HBV vaccine. After collection, serum samples were kept in a freezer and DBS were stored under two different conditions: standard (in a cool and dark place for 7 days) and hard (at room temperature for 7 days while outreach activities were conducted in Vientiane Municipality and Khammuan Province in central Lao PDR). The paired DBS and serum samples were then sent for testing to the Research Center for Hepatitis and Immunology, National Center for Global Health and Medicine, Ichikawa, Japan.

### Data entry and analysis

All completed questionnaires were sent to a centralized location where the data were double-entered and cross-checked using a Microsoft Excel 2007 spreadsheet. Data were analyzed using a linear regression model to compare the anti-HBs values from DBS and serum samples in STATA 14 (Stata Corp., College Station, TX).

### Ethical considerations

Our team members explained, both verbally and in writing, the objectives and procedures of the survey to the participants and local authorities. If a mother was illiterate, written informed consent was obtained from the father or a legal guardian. Obtaining informed consent, administering the questionnaire, collecting documented HBV vaccination histories, and drawing blood were all supervised. If a mother was < 16 years, written informed consent to participate in the study for themselves and for their child was obtained from a parent or a legal guardian. The study protocol was reviewed and approved by the ethics committees of the Ministry of Health, Lao PDR and the National Center for Global Health and Medicine, Japan (NCGM-950).

## Results

### Selection of participants

**Fig. 1:**
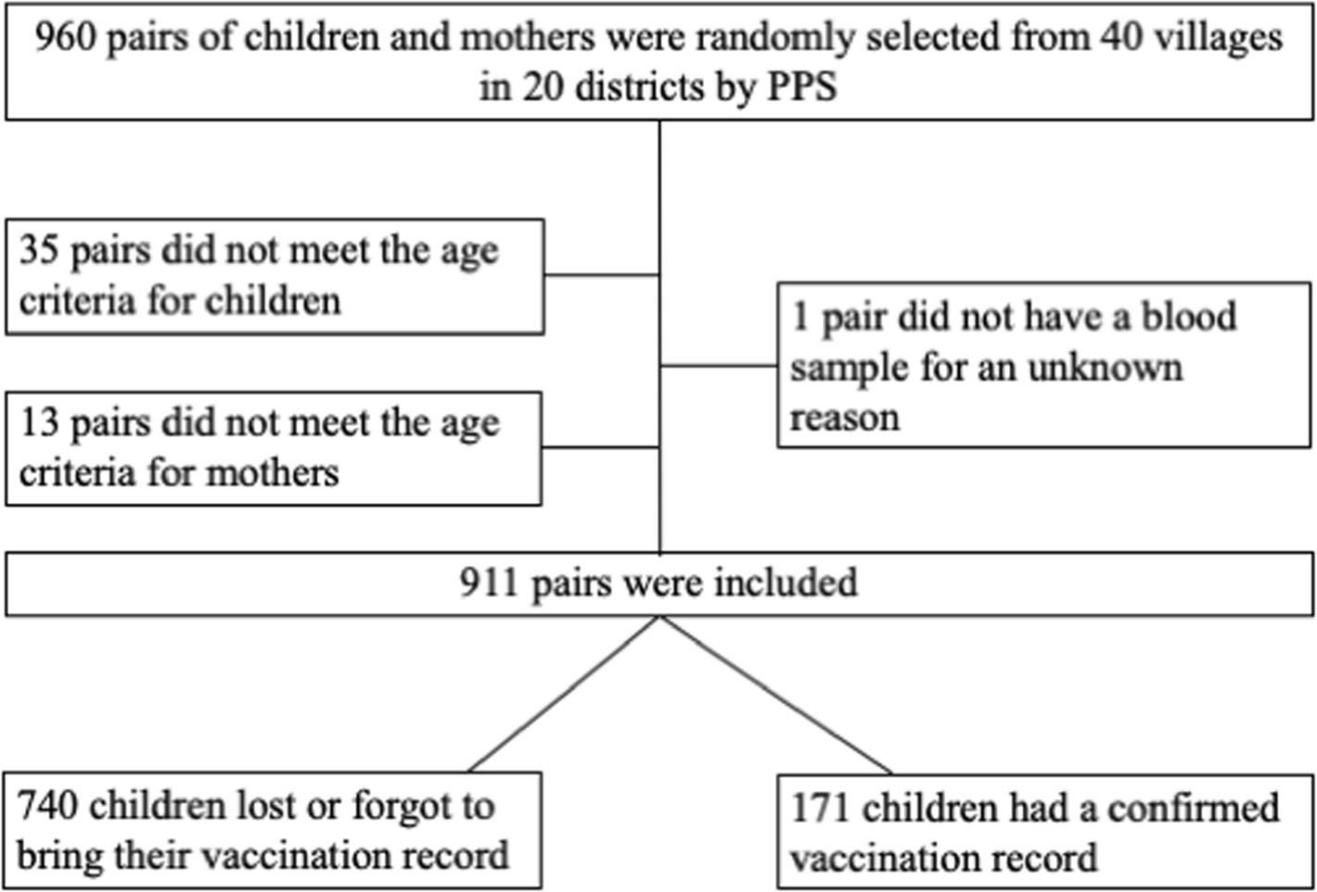
Flow diagram of the recruitment process. In probability proportional to size sampling, 960 pairs of children and mothers selected from 40 villages. After exclusion of 49 pairs, data for 911 pairs were analyzed in this study

Figure 1 is a flow chart of the recruitment of participants. Forty villages from 20 districts were randomly selected for inclusion by PPS. The survey team administered the questionnaires and performed blood sampling in all 40 villages. In total, 49 mother-child pairs were excluded from analysis: 35 did not meet the age requirement for the child (e.g., 3–4 years of age or unknown), 13 did not meet the age requirement for the mother (e.g., > 45 years of age or unknown), and one did not have a blood sample from the child (reason unknown). This left 911 mother-child pairs for analysis.

### Background characteristics

Table 1 shows the descriptive characteristics of the 911 children based on documented vaccination history. Among the 171 children who had a written immunization record, 42.9 and 11.6% were born in provincial and district hospitals, respectively. Among the 740 children who did not have a written immunization record, 63% were born at home. Most of the 911 mothers (85.9%) were from lowland Lao.

**Table 1:** Descriptive characteristics of children (3–9 years) after hepatitis B vaccination

Characteristic	Total (*n* = 911)		Undocumented vaccination history (*n* = 740)		Documented vaccination history (*n* = 171)							
					No doses (*n* = 10)		1 dose (*n* = 5)		2 doses (*n* = 9)		≥ 3 doses (*n* = 147)	
	n	(%)	n	(%)	n	(%)	n	(%)	n	(%)	n	(%)
Sex												
Male	453	(49.7)	379	(51.2)	4	(40.0)	3	(60.0)	1	(11.1)	66	(44.9)
Female	458	(50.3)	361	(48.8)	6	(60.0)	2	(40.0)	8	(88.9)	81	(55.1)
Birthplace												
Provincial hospital	205	(22.5)	133	(18.0)	3	(30.0)	2	(40.0)	4	(44.4)	63	(42.9)
District hospital	105	(11.5)	85	(11.5)	1	(10.0)	2	(40.0)	0	(0.0)	17	(11.6)
Health center	30	(3.3)	26	(3.5)	0	(0.0)	0	(0.0)	0	(0.0)	4	(2.7)
Private clinic	18	(2.0)	17	(2.3)	0	(0.0)	0	(0.0)	0	(0.0)	1	(0.7)
Home with visiting medical staff	535	(58.7)	467	(63.1)	6	(60.0)	1	(20.0)	5	(55.6)	56	(38.1)
At or near the home	18	(2.0)	12	(1.6)	0	(0.0)	0	(0.0)	0	(0.0)	6	(4.1)
Vaccination site												
Hospital	153	(16.8)	109	(14.7)	1	(10.0)	2	(40.0)	0	(0.0)	41	(27.9)
Health center	105	(11.5)	82	(11.1)	0	(0.0)	0	(0.0)	1	(11.1)	22	(15.0)
In the village with medical staff	276	(30.3)	233	(31.5)	2	(20.0)	0	(0.0)	4	(44.4)	37	(25.2)
Private doctor	6	(0.7)	4	(0.5)	0	(0.0)	0	(0.0)	0	(0.0)	2	(1.4)
Do not remember	6	(0.7)	5	(0.7)	0	(0.0)	0	(0.0)	0	(0.0)	1	(0.7)
Other	8	(0.9)	5	(0.7)	0	(0.0)	0	(0.0)	0	(0.0)	3	(2.0)
Not known/reported	357	(39.2)	302	(40.8)	7	(70.0)	3	(60.0)	4	(44.4)	41	(27.9)
Mother’s ethnicity												
Lowland Lao	783	(85.9)	635	(85.8)	10	(100.0)	5	(100.0)	8	(88.9)	125	(85)
Highland Lao	83	(9.1)	71	(9.6)	0	(0.0)	0	(0.0)	1	(11.1)	11	(7.5)
Hmong	5	(0.5)	5	(0.7)	0	(0.0)	0	(0.0)	0	(0.0)	0	(0.0)
Unknown	40	(4.4)	29	(3.9)	0	(0.0)	0	(0.0)	0	(0.0)	11	(7.5)

### Evaluation of DBS analysis

**Fig. 2:**
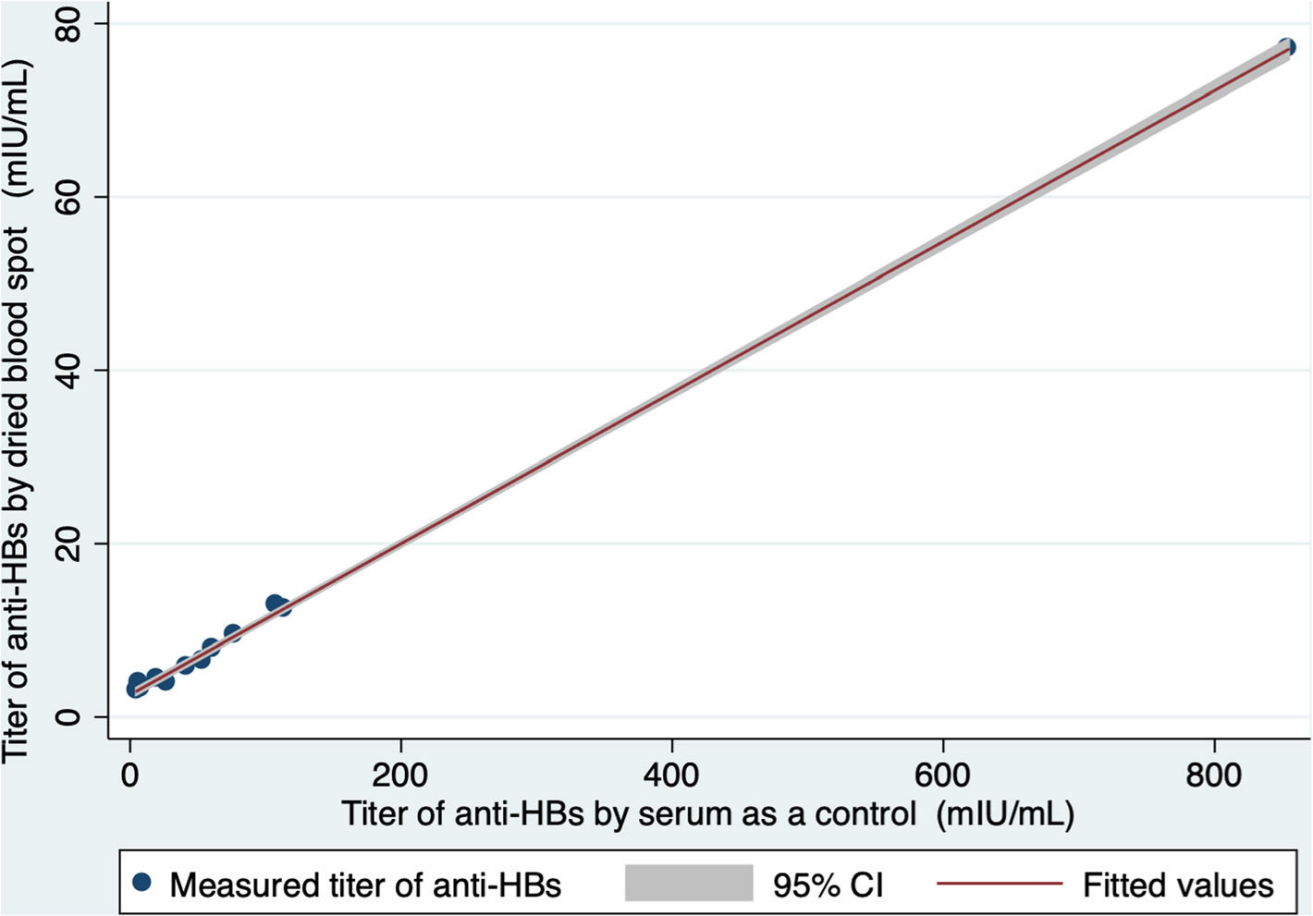
Anti-HBs values from dried blood spots (standard condition) vs. serum as a control. Linear regression shows a strong correlation between serum and DBS stored under standard conditions. Pearson’s correlation coefficient is 0.999

To evaluate the usefulness of the measured anti-HBs values by DBS, we compared values from the paired DBS and serum samples collected from 12 of 16 healthcare workers. Four participants were excluded from the analysis because serum values exceeded the detection range (> 1000 mIU/mL). There were no significant differences between DBS stored under standard and hard conditions (correlation coefficient = 0.999) as determined by linear regression analysis. Figure 2 shows a strong correlation between serum and DBS stored under standard conditions (correlation coefficient = 0.999), and the anti-HBs titers obtained from DBS were lower than those obtained from serum. Based on the linear regression model, 10 mIU/mL of serum anti-HBs is equivalent to 3.45 mIU/mL (95% CI: 3.06–3.85) of DBS anti-HBs.

### Anti-HBs and HBsAg among children and their mothers

Table 2 shows the serologic results of HBV infection according to number of vaccinations. Overall, 4.4% [95% CI: 3.0–5.7] (40/911) of the mothers and 2.3% [95% CI: 1.3–3.3] (21/911) of the children were HBsAg positive. Eleven out of 21 HBsAg positive children (52.3%) had HBsAg-positive mothers, whereas the other 10 were from non-infected mothers [6]. If all of 11 children were infected from their mothers, mother-to-child transmission rate can be calculated as 27.5% (11/40 = 27.5%). However, among the 147 children with a documented history of ≥3 doses of HBV vaccine, 7 of the mothers were HBsAg positive: 5 of their children were HBsAg negative and 2 were HBsAg positive (neither of 7 children had received a birth dose). None of these 147 children who received ≥3 doses were positive for anti-HBs with a cutoff value of 10 mIU/mL. When the cutoff value was reduced to 3.45 mIU/mL based on the DBS evaluation result, one child (0.7%) was positive for anti-HBs. When the cutoff value was reduced to 1.5 mIU/mL based on another study using DBS testing with Grade 903 filter cards [11], 6 children (4.1%) were positive for anti-HBs.

**Table 2:** Results of hepatitis B serology among 911 mother- child pairs in central Lao PDR

Result of hepatitis B serology	Total (*n* = 911)		Undocumented vaccination history of child (*n* = 740)		Documented vaccination history of child(*n* = 171)							
					No doses (*n* = 10)		1 dose (*n* = 5)		2 doses (*n* = 9)		≥ 3 doses (*n* = 147)	
	n	(%)	n	(%)	n	(%)	n	(%)	n	(%)	n	(%)
Mother HBs^a^ antigen positive	40	(4.4)	32	(4.3)	0	(0.0)	0	(0.0)	1	(11.1)	7	(4.8)
Children HBs^a^ antigen positive	21	(2.3)	19	(2.6)	0	(0.0)	0	(0.0)	0	(0.0)	2	(1.4)
Children anti-HBs^a^ positive												
Cutoff value > 10 mIU/mL	6	(0.7)	6	(0.8)	0	(0.0)	0	(0.0)	0	(0.0)	0	(0.0)
Cutoff value > 3.45 mIU/mL^b^	12	(1.3)	11	(1.5)	0	(0.0)	0	(0.0)	0	(0.0)	1	(0.7)

^a^Hepatitis B surface

^b^Cutoff value was decided from our linear regression model

## Discussion

### Results overview

In this cross-sectional study measuring anti-HBs among randomly selected children in Lao PDR, our three major findings were as follows. First, positivity for anti-HBs was extremely low compared with that reported previously, with only one of 147 children with a confirmed vaccination history of ≥3 HBV doses reaching a positive level when applying the DBS cutoff value of 3.45 mIU/mL. Second, 2 children without a birth dose from HBsAg-positive mothers acquired chronic HBV infection despite receiving 3 doses during infancy. Third, the results from the DBS samples correlated well with those from the serum samples.

Anti-HBs positivity.

We targeted children aged 5–9 years, which meant that 3–9 years had passed since they received their last HBV vaccination. Based on a literature review of 43 previous studies [12], if measured within 1 year after a completed vaccination series, there would be a 98% (range, 52–100%) median positivity rate for anti-HBs with anti-HBs levels ≥10 mIU/mL after 3 or 4 doses of vaccine in the population studied. After receiving 3 doses of vaccine, anti-HBs titers decrease gradually and the positivity rate for anti-HBs varies from73.4% [13] and 95% [14] at 3 years after last vaccination to 40.1% [15], 44.8% [13], 65.2% [14], and 66.7% [16] at 10 years after last vaccination. In a cohort study of 15 Alaskan communities, 51% of subjects were still serologically protected 30 years after receiving 3 doses of plasma-derived HBV vaccine [17]. In our study, at 3–9 years after last vaccination, none of the participants showed serologic protection at the cutoff of 10 mIU/mL and only one (0.7%) reached > 3.45 mIU/mL for the DBS cutoff. This positivity rate is thus extremely low compared with other studies. Specifically compared with previous studies conducted in Lao PDR, such as Huaphan, a serologic investigation that used non-random sampling of participants with at least 3 documented HBV vaccinations revealed that 17.0% (15/55) were serologically protected (> 10 mIU/mL by serum) at the age of 1–4 years [18]. Another study with 3 documented DTP-Hepatitis B-Hib vaccinations in Vientiane, Khammouane, and Boulhikhamxay provinces in Lao PDR revealed 37.9% (394/1039) of participants were serologically protected (> 10 mIU/mL by serum) at the age of 9–50 months [19]. Thus, unexpectedly low seropositive rates were found among Laotian children with a documented vaccination history in both of these serologic studies [18, 19] nearly as bad as in our study. Our extremely low rate relative to these two studies may be because we investigated a random sample of children, whereas the two previous serologic investigations investigated non-randomized population samples as well as had differences in study design and methodology, and may also reflect the reality of the situation in hard-to-reach areas where observed cold chain problems can result in inactive vaccine immunization. However, further investigations are needed to confirm this speculation.

Problems in cold chain transportation and storage might be a key reason for the extremely low positivity rate found in the present study. Appropriate cold chain transport can be difficult to maintain in developing countries and can thus reduce the effectiveness of vaccination programs. Lao PDR is a tropical country, and we have observed the inadvertent freezing of vaccine samples by the use of ice packs during transportation. HBV vaccines are sensitive to freezing, which causes the HBsAg protein to dissociate from the aluminum adjuvant and thereby lose immunogenicity and potency. Vaccines containing aluminum salt adjuvant damaged by freezing represent a real risk to the effectiveness of immunization programs [21]. Such problems have been reported from colder countries such as Mongolia, where 19% of 181 provincial-to-rural transports (95% CI: 13–25%) resulted in freezing [22]. Moreover, refrigeration for storage is not always easy to control precisely enough. Our pilot study in Lao PDR also revealed that the vaccines were sometimes exposed to freezing during storage at district level or during transportation [8]. Most serologic studies tend to be conducted in easy-to-access areas, but because the present study selected subjects randomly using PPS from central Lao PDR that includes hard-to-access areas, it is possible that vaccines were inactivated due to cold chain problems. The target population was 5 to 9 years old in January 2011 and therefore the HB vaccine series was completed between 2002 and 2006 when cold chain and other EPI services were not as good as in recent years.

Our second main finding is also evidence of the ineffectiveness in the HBV vaccination program. From the 147 children who received ≥3 doses of the HBV vaccine, 2 children who were born to mothers that were HBsAg positive acquired chronic HBV infection vertically during birth or horizontally later in early childhood. We recognize that this is a very small sample with just a total of 7 HBsAg-positive mothers, but the rate (2/7 = 29%) of transmission does seem to be higher than that of vaccinated population. In a meta-analysis of four trials [20], relative risk of HBV infection from an HBV-positive mother was 0.28 (95% CI: 0.20–0.40), with HBV infection occurring in 33 of 252 (13%) vaccinated children and 77 of 151(51%) unvaccinated children. The latter rate of HBV infection in unvaccinated children is similar to that for our vaccinated children. The infection rate from present study among vaccinated children (29%) stands between that of the vaccinated and unvaccinated population, and therefore the HBV vaccination program does not seem to be effective in Lao PDR.

### Correlation between serum and DBS results

Lastly, a strong correlation was found between serum and DBS results in this study, although the anti-HBs titers obtained from the DBS samples were lower than those obtained from the serum samples. In another study comparing anti-HBs values obtained from serum samples with the values obtained using the same DBS method as used in the present study, the false-negative rate was 14.2% (47/331) when using a cutoff value of 10 mIU/mL for the DBS, with optimal discrimination was achieved with a cutoff value of 1.5 mIU/mL (sensitivity, 0.917; specificity, 0.993) [11]. In the present study, when applying the cutoff value of 1.5 mIU/mL, only 6 children (4.1%) were positive for anti-HBs (Table 2). This rate is still extremely low compared with other studies [12–17], and again suggests problems with the effectiveness of Lao PDR’s HBV vaccination program. DBS testing has lower sensitivity than serologic testing and there is no consensus yet as to the cutoff level to be used for serologic protection. This warrants further research because DBS could be a low-cost solution to evaluate the effectiveness of vaccination programs, especially in hard-to-reach areas, and thus could contribute to efforts to provide universal immunization program.

### Limitations

This study has some limitations. The Centers for Disease Control and Prevention in the United States recommend performing post-vaccination serologic testing 1–2 months after the final dose during infancy [23, 24] because antibody titers decrease over time. Furthermore, our study did not evaluate anamnestic reactions, including following a booster vaccination, for participants with extremely low anti-HBs. In a previous study, over 90% of vaccinated participants with an anti-HBs level < 10 mIU/mL showed an anamnestic response, with anti-HBs levels ≥10 mIU/ml after the HBV booster dose [23, 24]. Therefore, further investigations should include post-vaccination serologic testing performed 1–2 months after the final dose of vaccine or booster vaccination in order to evaluate anamnestic reactions. A second limitation of the present study was it cross-sectional design, which does not allow conclusions about cause and effect to be drawn. The causes of the extremely low response to HBV vaccination found among children in central Lao PDR require further study, including investigation of the adequate vaccine transportation and storage. Third, we did not investigate potential factors which may influence the anti-HBs positivity in vaccinated children, including malnutrition and HIV infection. The positivity rate for anti-HBs varies depending on host factors. Globally, malnutrition has been described as the most common underlying condition of immunodeficiency, which suggests that malnourished children may not be able to respond to vaccines effectively [25]. Regarding HBV vaccination, there is controversial data regarding whether malnutrition influences the seroresponse for anti-HBs. In a study in Guatemala, the positivity rate for anti-HBs was almost the same between Latino, Native Indian, and malnourished participants at the age of 12 months (96.2, 98.4, and 100%, respectively) [26]. On the other hand, in a study in Egypt, 100% of healthy infants (geometric mean titer of 135.23 ± 28.44 mIU/mL) and 87% (27 of 31) of infants with protein-calorie malnutrition (geometric mean titer of 98.75 ± 44.68 mIU/mL) were positive for anti-HBs at the age of 8 months [27]. In addition, HIV infection reduces the response to vaccination. In one study, patients who were not infected with HIV had significantly higher serological response rates than those who were in a retrospective cohort (70% versus 41.1%; RR: 0.586, 95% CI: 0.36–0.96) [28]. In a meta-analysis regarding long-term immune response among HIV patients, the positivity rate for anti-HBs tended to decrease over time: after three doses of HBV vaccine, 71% of primary responders maintained protective antibody titers at year 1, 33–61% at year 2, and 40% at year 5 [29]. In the present study, we did not collect any information regarding host factors, including indicators for malnutrition, low birth weight, or HIV infection. Considering the malnutrition rate (the prevalence of stunting is 48% in children under 5 years of age) [30] and low HIV prevalence (0.2% among adults) [31] in Lao PDR, the present study may slightly underestimate anti-HBs positivity rates among children who received the HBV vaccine. Fourth, we cannot evaluate the quality of the vaccines given because the manufacturer name and lot number are not usually recorded on the children’s immunization certificates. Vaccines are mainly procured by partners; only an average of around 7% of total vaccines were purchased with state funds between 2004 and 2014 [32]. Thus, vaccine manufacturers are determined by the partners, and the government does not keep accurate, up-to-date information on the vaccine manufacturers. Further investigations are needed to evaluate the quality of vaccine used. Lastly, our study included only 12 healthy volunteers, which was not enough to evaluate the usefulness of DBS. While the correlation coefficient was satisfactory at 0.999 (Fig. 2.), further studies of a large number of samples are required.

## Conclusions

Anti-HBs positivity rates were much lower than expected in a randomly selected population with ≥3 doses of documented HBV vaccination in central Lao PDR. Vaccination coverage is widely used as an indicator of the effectiveness of vaccination programs, and most countries and international organizations try to increase vaccination coverage. However, the present findings reveal that the nationwide HBV vaccination program did not confer immunity under certain conditions. The results highlight the importance of post-vaccination serologic testing to determine whether vaccines provide adequate protection against HBV, which cannot be drawn from the conventional indicator of immunization coverage only. Currently WHO does not recommend conducting individual post-vaccination serologic testing [2], however, it is recommended to test the population effectiveness. The standardization of DBS testing and determination of an appropriate cut-off value is needed, because problems with cold chain transportation and storage will likely continue in developing countries, in hard-to-reach areas especially, and DBS testing is a potential low-cost solution to evaluating the effectiveness of HBV vaccination programs. Future studies using post-serological testing should use randomization to determine the reality of program effectiveness especially in developing countries.

## Abbreviations

DBS: Dried blood spots
HBs: Hepatitis B surface
HBsAg: Hepatitis B surface antigen
HBV: Hepatitis B virus
PPS: Probability-proportional-to-size
RLU: Relative light unit
RR: Relative risk
